# CO_2_ Sorption on Ti-, Zr-, and [Ti,Zr]-Pillared Montmorillonites

**DOI:** 10.3390/ma17164036

**Published:** 2024-08-14

**Authors:** Agnieszka Klimek, Adam Gaweł, Katarzyna Górniak, Anna Tomczyk-Chmiel, Ewa M. Serwicka, Krzysztof Bahranowski

**Affiliations:** 1 Faculty of Geology, Geophysics and Environmental Protection, AGH University of Science and Technology, al. Mickiewicza 30, 30-059 Krakow, Poland; aklimek@agh.edu.pl (A.K.); agawel@agh.edu.pl (A.G.); gorniak@agh.edu.pl (K.G.); tomczyk.an@gmail.com (A.T.-C.); 2 Jerzy Haber Institute of Catalysis and Surface Chemistry, Polish Academy of Sciences, Niezapominajek 8, 30-239 Krakow, Poland; ncserwic@cyf-kr.edu.pl

**Keywords:** CO_2_ sorption, pillared montmorillonites, STA CO_2_-QMS

## Abstract

Montmorillonite is a layered clay mineral whose modification by pillaring, i.e., insertion of oxide nanoclusters between the layers, yields porous materials of great potential in sorption and catalysis. In the present study, an unrefined industrial bentonite from Kopernica (Slovakia), containing ca. 70% of montmorillonite, was used for the preparation of Ti-, Zr-, and mixed [Ti,Zr]-pillared clay sorbents. The pillared samples were characterized with X-ray diffraction (XRD), field emission scanning electron microscopy (FESEM), and N_2_ adsorption at −196 °C and tested for the capacity of CO_2_ sorption at 0 °C and 1 bar pressure. The experiments revealed that pillared samples sorbed at least four times more CO_2_ than the parent bentonite. Of the materials tested, the sample pillared with mixed [Ti,Zr] oxide props showed the best performance, which was attributed to its superior microporosity. The results of CO_2_ adsorption demonstrated that the cost-effective use of crude industrial bentonite as the sorbent precursor is a viable synthesis option. In another experiment, all pillared montmorillonites were subjected to 24 h exposure at room temperature to a flow of dry CO_2_ and then tested using simultaneous thermal analysis (STA) and the mass spectrometry (MS) analysis of the evolving gases (STA/QMS). It was found that interaction with dry CO_2_ reduces the amount of bound carbon dioxide and affects the processes of dehydration, dehydroxylation, and the mode of CO_2_ binding in the pillared structure.

## 1. Introduction

Montmorillonites are layered silicates of 2:1 structure, i.e., layers are built of two tetrahedral, silicon–oxygen sheets that sandwich one octahedral, metal–oxygen–hydroxyl sheet. The layer has a negative charge that is neutralized by the interlayer, usually hydrated, cations [1]. Montmorillonites, both in their crude form and after physical and/or chemical modification, find a range of practical applications, from the production of drilling mud or foundry sand to the preparation of catalysts and pharmaceuticals [2].

**Figure 1:**
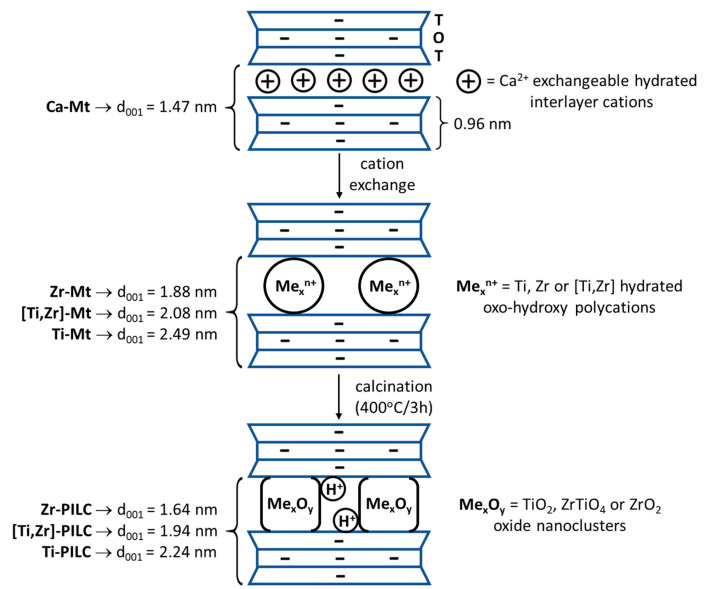
Schematic illustration of pillaring montmorillonite with Ti-, Zr-, and mixed [Ti,Zr]-polycations.

Chemical modifications of montmorillonites most commonly used on a technical scale include treatment with sodium salts (e.g., sodium carbonate) or inorganic acids (e.g., sulfuric acid) [3,4]. As to laboratory scale modifications, a procedure referred to as pillaring, which has been known for more than half a century, belongs to the most researched topics [5,6]. The synthesis of pillared montmorillonites, referred to as PILC (pillared interlayered clay), is a type of material engineering at the atomic level, which leads to fundamental changes in the structure of montmorillonite and its physicochemical properties. During pillaring, simple hydrated cations such as Na^+^ or Ca^2+^ present in the interlayer spaces of montmorillonites are replaced with large, hydrated, oxo-hydroxy polycations, most often Al, Ti, or Zr [5]. Such replacement increases the interlayer spacing, and props open the structure of the mineral. Subsequent calcination at temperatures around 400 °C results in the transformation of hydrated hydroxypolycations into metal oxide pillars that firmly bind adjacent layers [5]. The layer charge in pillared montmorillonite is compensated by protons remaining in the structure after the dehydroxylation of polycations [5]. Due to the increased interlayer spacing and spatial separation of pillars, PILC materials are micro/mesoporous structures of high specific surface area (usually in the range of 100–400 m^2^/g [5,7]), easily accessible to gases, vapors, and liquids. For this reason, the sorption properties of pillared montmorillonites have been widely investigated [5,6,7]. The pillaring process and its effect on the structural and compositional characteristics of montmorillonite is schematically illustrated in Figure 1, using the materials studied in this work as an example.

Today, with the existential threat of climate change, CO_2_ capture is a research topic of great importance [8]. Analysis of recent reviews and case studies addressing this issue shows that various micro- and mesoporous materials (e.g., zeolites, mesoporous molecular sieves, or activated carbons) have been tested as potential CO_2_ sorbents [9,10,11,12,13,14,15,16,17]. There are also publications on the use of raw or modified clay minerals for this purpose, in particular, pillared montmorillonites, with most of them dealing with the properties of Al-PILC materials [18,19,20,21,22,23,24,25,26,27,28,29,30,31]. Works on montmorillonites pillared with Ti or Zr oxide nanoclusters are less numerous, and the results either vary significantly or are too few to compare. For instance, the CO_2_ sorption capacity reported for Zr-PILC ranges from ca. 0.5 mmol/g [18] to 0.8 mmol/g [23] to 1–1.5 mmol/g [20]. CO_2_ capture by Ti-PILC was reported in two articles, but the results are incomparable due to different experimental conditions [18,19]. As for the mixed [Ti,Zr]-pillared montmorillonite, which has been shown to represent a new quality with respect to Ti- or Zr-PILC [32,33,34], there are no data available on its CO_2_ sorption capacity. The present work seeks to fill this gap and deepen the understanding of CO_2_ interaction with pillared clays by investigating the sorption of CO_2_ on a series of montmorillonites hosting in their interlayer spaces Ti, Zr, and Ti–Zr pillars. Simultaneous thermal analysis with quadrupole mass spectrometry analysis (STA QMS) of gaseous products enabled quantitative estimation of bound CO_2_ and the temperatures at which this gas is removed from the surfaces of heated PILCs. In addition, the study addresses the influence of CO_2_ sorption on the content of water of hydration and the amount of structural hydroxyls in PILC samples. At variance with previous studies, pillared materials were prepared from a crude industrial bentonite used without any refining pretreatment.

## 2. Materials and Methods

**Figure 2:**
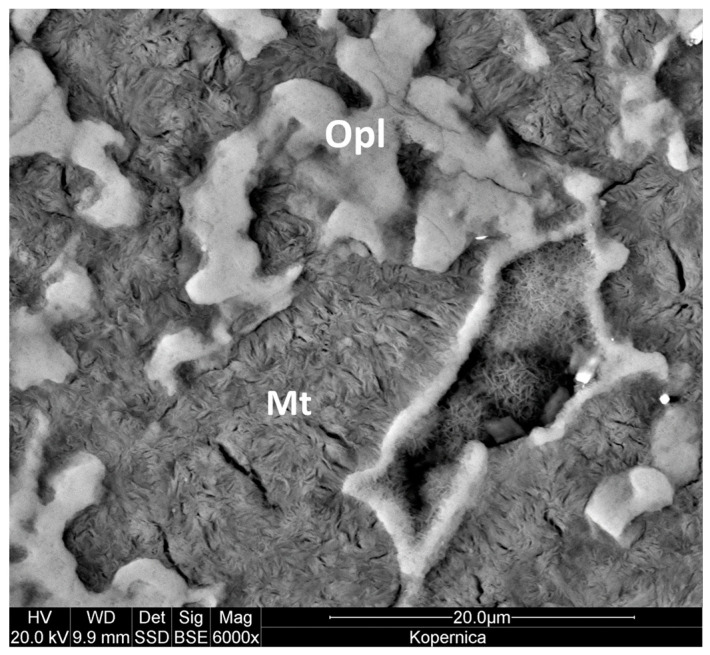
FESEM/BSE image of the polished Kopernica bentonite sample showing montmorillonite (Mt) lumps embedded in opal C/CT (Opl) (areas of greater brightness). For details on mineral identification, see [35].

The study was carried out on wholesale industrial bentonite from the Kopernica deposit in Slovakia, consisting of ~70 wt.% of Ca-montmorillonite, ~17 wt.% of opal C/CT, ~5 wt.% of quartz, ~5 wt.% of biotite, and ~3 wt.% of feldspars, as determined in our previous work [35]. We have shown that the average mineral composition of Kopernica bentonite is not significantly different from that of its clay fraction (i.e., less than 2 µm) due to the cementation of montmorillonite crystals with opal (Figure 2), which hinders the separation of smectite from the bentonite rock. For this reason, Kopernica bentonite was used without any further pretreatment. The pillaring procedure was described in detail previously [32]. Briefly, three pillaring agents were prepared. A 0.1 M aqueous solution of ZrOCl_2·_8H_2_O was used as a Zr-pillaring agent. A Ti-pillaring solution was obtained by acid hydrolysis of TiCl_4_. The Zr–Ti-pillaring agent was obtained by mixing the Ti- and Zr-pillaring solutions in a 1:1 molar ratio. Subsequently, an appropriate pillaring solution was added to the bentonite powder in an amount corresponding to 10 mmol of pillaring cations per 1 g of Ca-montmorillonite, and the vigorously stirred suspension was aged for 1 h at 40 °C. The resulting precipitate was washed with distilled water until free of chloride ions, dried in a drying box at 50 °C, and finally calcined at 400 °C for 3 h. The prepared materials are further referred to as Zr-Mt, Ti-Mt, and [Ti,Zr]-Mt for uncalcined samples, and Zr-PILC, Ti-PILC, and [Ti,Zr]-PILC for calcined powders. The parent raw material is denoted Kopernica.

X-ray fluorescence (XRF) spectroscopy was used to determine the chemical composition of the samples. The analysis was carried out with a ZSX Primus II (Rigaku, Tokyo, Japan) spectrometer (Rh anode) using a calibration based on the certified reference materials.

The powder X-ray diffraction (XRD) patterns were obtained using a Rigaku SmartLab diffractometer (Rigaku Corporation, Tokyo, Japan) under the following conditions: graphite-monochromatized CuKα radiation, operating voltage = 45 kV, current = 200 mA, step size = 0.05°2θ, and counting time = 1 s/step.

Field emission scanning electron microscopy (FESEM) studies were carried out with an FEI Quanta 200 FEG SEM (Fei, Hillsborough, OR, USA) and, alternatively, with a JSM7500F (JEOL, Tokyo, Japan) scanning electron microscopes equipped with secondary electron (SE) and backscattered electron (BSE) detectors. Uncoated polished thin sections of samples impregnated with epoxy resin (BSE imaging) and uncoated powdered samples (SE imaging) were analyzed. Imaging modes and instrument conditions are given on the data bars of the individual images.

Textural parameters of investigated samples were determined from nitrogen adsorption/desorption isotherms at –196 °C, with an ASAP 2020 (Micromeritics, Norcross, GA, USA) apparatus under relative pressures from 10^−3^ to 0.99. Before measurement, each sample was outgassed at 150 °C for 12 h. BET formalism was used for the calculation of specific surface areas (SBET). The total pore volume (Vtot) was determined from the amount of N2 adsorbed at p/p0 = 0.99. Mesopore volume (V_meso_) was determined from the adsorption branch using the BJH method, while micropore volume (V_micro_) was calculated using Dubinin–Radushkevich method. The volume of macropores V_macro_ was determined by subtracting the volume of mesopores and micropores from the total pore volume (V_tot_ − (V_meso_ + V_micro_)).

The CO_2_ sorption capacity of pillared montmorillonites was evaluated using the same ASAP 2020 instrument, by measuring the amounts of adsorbed CO_2_ at 0 °C temperature, within the range of relative pressures p/p_0_ = 0.001–0.03, after previous outgassing of the samples at 150 °C for 12 h.

Interaction between the investigated materials and CO_2_ was also conducted in the experimental set-up involving exposure of the sorbent to CO_2_ flow at room temperature. In this experiment, 100 mg of pillared montmorillonite sample was scattered onto the Petri dish and placed in the Paar reactor for 24 h under the flow of dry CO_2_ (80 mL/min). Then, the montmorillonite sample was quickly transferred to Netzsch STA 449 Jupiter F5 apparatus (Netzsch, Selb, Germany) coupled with Netzsch QMS 403D Aeolos quadrupole mass spectrometer (Netzsch, Selb, Germany) for simultaneous thermal analysis (STA) (TGA-DTG-DTA) and the mass spectrometry (MS) analysis of the evolving gases. The experimental conditions were as follows: heating rate 20 °C/min, He flow 50 mL/min, and sample mass ∼30 mg. The amount of CO_2_ released from the analyzed sample was determined from the area beneath the QMS CO_2_ curve using the calibration curve prepared by analysis of CO_2_ emission from weighted samples of analytically pure calcium carbonate.

## 3. Results and Discussion

### 3.1. Chemical Analysis

The results of chemical analysis by XRF are given in Table 1. As expected, the pillaring resulted in enrichment of the starting material in titanium and zirconium oxides. At the same time, the modification led to an almost complete loss of CaO, as Ca^2+^ cations, present as charge-compensating cations in the montmorillonite interlayer, were exchanged during the pillaring process.

### 3.2. XRD Analysis

**Figure 3:**
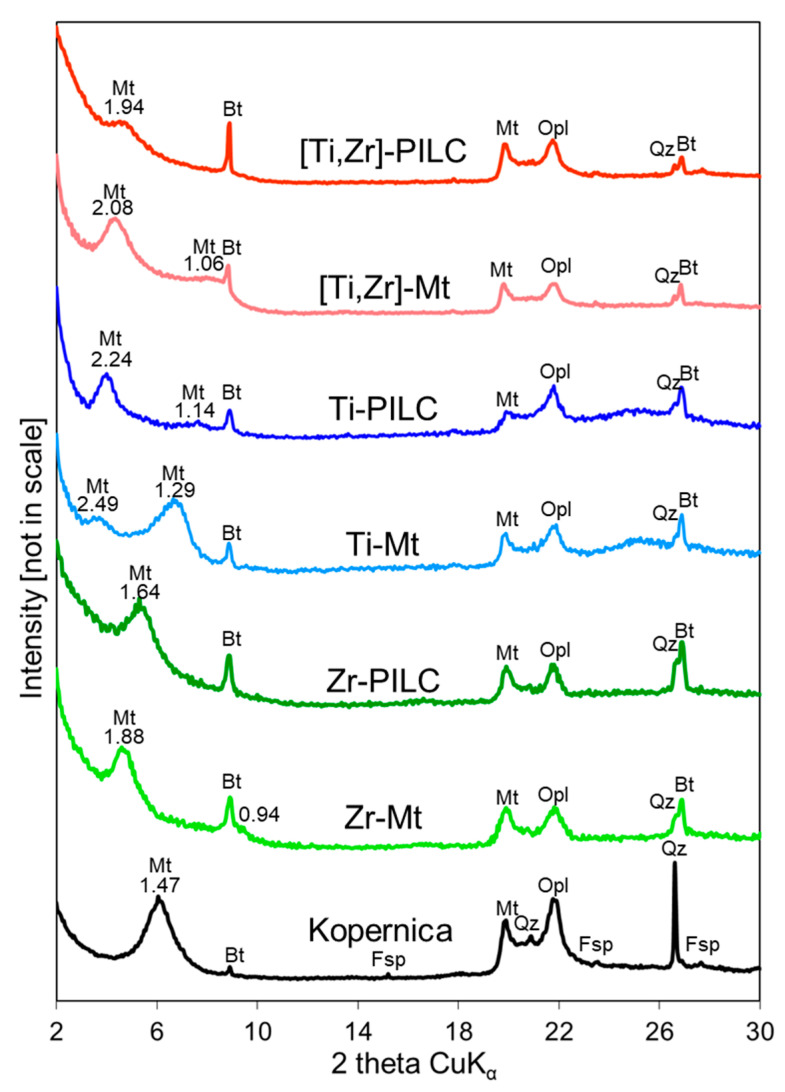
XRD patterns of the parent Kopernica material and samples obtained by modification with Zr, Ti, and [Ti,Zr] pillaring agents before and after calcination at 400 °C (Mt—montmorillonite, Bt—biotite, Opl—opal, Fsp—feldspar, Qz—quartz).

Figure 3 shows the XRD patterns of the parent Kopernica material and samples obtained by cation exchange with Zr, Ti, and [Ti,Zr] pillaring agents before and after calcination at 400 °C. The positions of 001 reflections, the corresponding FWHM index, and interplanar distances are listed in Table 2.

In accordance with the previous mineralogical studies [35], the untreated Kopernica sample is polymineral, with an XRD pattern dominated by reflections of Ca-montmorillonite (d_001_ = 1.47 nm, ref. code 13-135). In addition, reflections of biotite (ref. code 01-088-1899), opal, quartz (ref. code 46-1045), and feldspar (albite, ref. code 09-0466) are also visible. In all cases, the pillaring of montmorillonite was successful, as evidenced by increased values of interlayer spacings derived from the basal 001 reflection (Table 2, samples denoted Mt). In all diffractograms, 002 reflections are also observed (0.94, 1.29, and 1.06 nm, for Zr-Mt, Ti-Mt, and [Ti,Zr]-Mt, respectively, Figure 3). After calcination-induced dehydration/dehydroxylation of the pillars, the interlayer spacings decrease slightly (Table 2, samples denoted PILC). Table 2 also provides information on the FWHM (full width at half maximum) of the 001 reflection. The larger the FWHM, the smaller the coherently scattering structural domains along the [001] axis. The data in Table 2 show that pillaring appears to increase the long-range ordering in the *c* direction. A similar observation has been made by González et al. [36], who studied a series of Al-pillared montmorillonites. In the present work, this effect is attributed to the texturing of clay mineral particles caused by washing and drying associated with the pillaring of montmorillonite. It has been shown that wet clay tends to self-order when dried in a drying box due to the occurrence of sedimentation phenomenon, which enhances the horizontal orientation of platy clay particles [37]. The effect of calcination on FWHM varied from sample to sample. Among the factors that can affect the thickness of coherently scattering domains, calcination-induced dehydration of interlayer oligocations, which is different in each case, can be considered the main reason for the observed differences. The pillaring experiments are graphically summarized in Figure 1.

### 3.3. Textural Analysis

**Figure 4:**
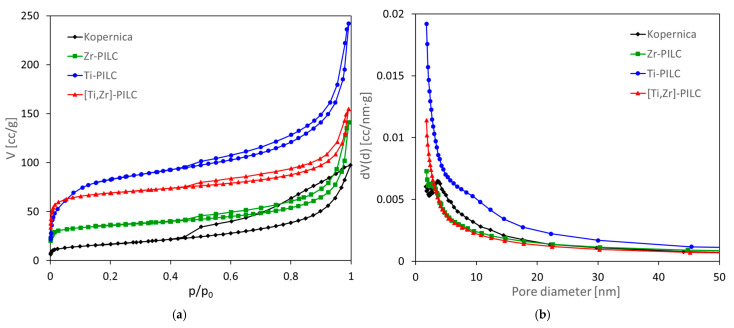
Textural analysis of investigated materials: (**a**) N_2_ adsorption/desorption isotherms at −196 °C and (**b**) pore size distribution curves.

Textural properties of the investigated pillared clays, i.e., specific surface area and porosity, are of particular importance for understanding the material’s ability to capture CO_2_ [38]. Therefore, the parent Kopernica montmorillonite and all PILC samples were subjected to standard textural examinations by means of nitrogen adsorption/desorption at −196 °C. The resulting isotherms are presented in Figure 4a. All isotherms of pillared clays lay above that of the starting montmorillonite, indicating enhanced sorption capacity, increasing in the order Zr-PILC < [Ti,Zr]-PILC < Ti-PILC, in accordance with the previous findings [32]. The isotherms of Kopernica, Zr-PILC and [Ti,Zr]-PILC are of mixed type I(a)/type II character. At low p/p_0,_ they can be described as type I(a), with a nearly horizontal course and a steep initial N_2_ uptake, characteristic of solids with narrow micropores of less than ca. 1 nm width [39]. The upward swing toward p/p_0_ = 1, indicating unrestricted multilayer adsorption, is a feature of type II isotherm. The Ti-PILC isotherm also shows a mixed character, but its part at low p/p_0_ shows a less steep rise, characteristic of type I(b) isotherm, occurring in materials with wider micropores overlapping with narrow mesopores of less than 2.5 nm diameter [39]. Indeed, the pore size distribution curves in the mesopore range, derived from the recorded isotherms, show that mesoporosity is particularly well developed in Ti-PILC and that it is dominated by small pores with diameters close to the lower limit of mesoporosity. All isotherms exhibit H4-type hysteresis loops, narrow in the case of pillared samples and broad in the case of the parent Kopernica montmorillonite, determined by the presence of platy particles and slit-shaped pores.

The textural parameters derived from the isotherms are presented in Table 3.

The raw bentonite sample from the Kopernica deposit showed the lowest value of the specific surface area of all the samples tested. It should be noted that fine-grained (µm or less) montmorillonite is the component determining the specific surface area of Kopernica. Clasts of biotite, feldspars, and quartz, known to be nonporous, are much larger (up to 1 mm); hence, their influence on the specific surface area of bentonite and its sorption properties is relatively low. It is clear that the pillaring process increased the specific surface area several times. The effect was attributed to the increased interlayer distances, which made the inner surfaces of montmorillonite and surfaces of pillars accessible for nitrogen sorption. As can be seen from Figure 1, the pillar height may be estimated by subtracting the layer thickness (taken as 0.96 nm) from the interlayer distance d_001_. The calculation shows that Ti pillars were the highest (ca. 1.28 nm), Ti–Zr pillars were somewhat lower (ca. 0.98 nm), and Zr pillars were the lowest (ca. 0.69 nm). The specific surface areas decrease in the same order (Table 3), indicating that pillar height plays an important role in shaping the specific surface area of PILC samples.

Another important factor is porosity and the share of specific types of pores, especially micropores, in the overall volume of the pore network [40]. The porosity in montmorillonite can originate from the interlayer space of primary crystallites, from the area of contact between the primary crystallites stacked within the flake-like aggregates, or from the interaggregate contacts [41]. The pores formed in the interlayer are usually of less than 2 nm width; hence, they are classified as micropores. The interparticle pores in flake-like aggregates are predominantly mesoporous (pore width between 2 and 50 nm), while the interparticle voids between the aggregates form macropores (pore width more than 50 nm). The raw Kopernica sample is predominantly meso- and macroporous, with the micropores contributing only 12% to the total pore volume. The main effect of pillaring is the generation of microporosity because pillars open the interlayer for adsorption. The largest relative share of micropores (over 50% of total pore volume) was observed in montmorillonite with mixed Ti–Zr pillars, compared to ~33% in Ti-PILC and only ~26% in Zr-PILC. The enhanced microporosity of the PILC samples affected accordingly the average pore diameters so that their values decreased in the order: Kopernica > Zr-PILC > Ti-PILC > [Ti,Zr]-PILC (Table 3). Mesopores also represent an important part of the pore network in pillared samples (Figure 4b, Table 3). Their existence facilitates the access of gas molecules to the micropores and thus enhances the overall sorption properties of PILC.

### 3.4. FESEM Analysis

**Figure 5:**
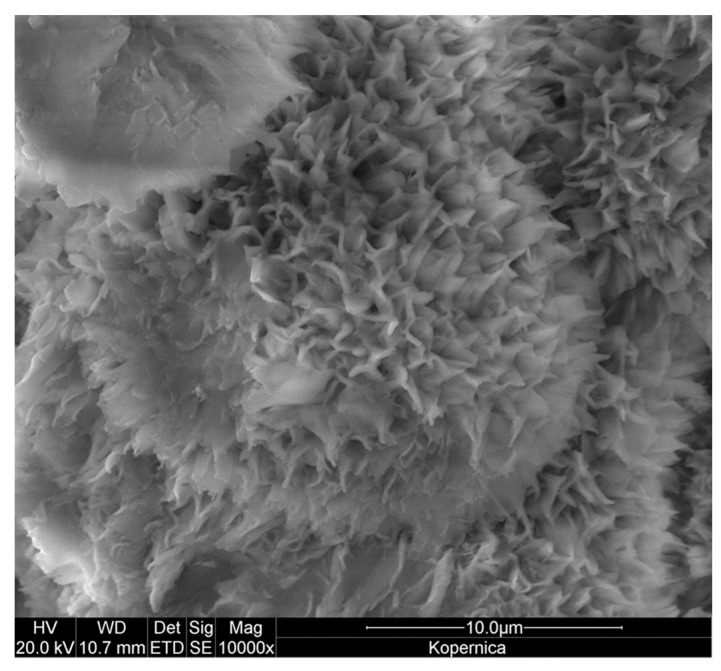
FESEM SE image of Kopernica bentonite sample with rose-like clusters of montmorillonite flakes.

The FESEM image in Figure 5 shows the morphology of the Kopernica sample. The shape of montmorillonite grains reflects its layered crystalline structure, as aggregates of primary lamellar crystallites form flake-like particles. Individual flakes are arranged in large rose-like clusters with an open texture and micrometer-sized voids between the flakes. This system of interparticle macropores facilitates the diffusion of gases and vapors in the clay mineral. On the other hand, micro- and mesopores, identified by N_2_ sorption at −196 °C, are not revealed in the FESEM examination, as they are part of the internal microstructure of the flake-like particles.

**Figure 6:**
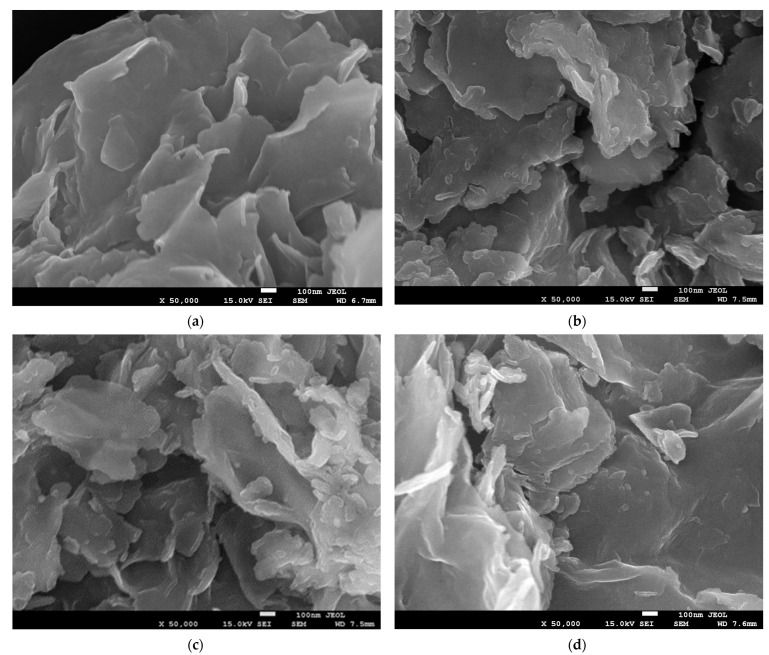
FESEM SE images of (**a**) Kopernica montmorillonite, (**b**) Zr-PILC, (**c**) Ti-PILC, and (**d**) [Ti,Zr]-PILC.

Figure 6 provides a comparison of SEM images of the parent montmorillonite and its pillared derivatives. It is clear that although the pillared samples retain a flake-like morphology, the particles appear to be more agglomerated and partially crushed. Both effects can be attributed to the thermal pretreatment that the pillared samples underwent. Sedimentation occurring during the drying of the wet cake and shrinking upon subsequent calcination is considered the cause of the observed evolution of morphology.

### 3.5. CO_2_ Sorption

**Figure 7:**
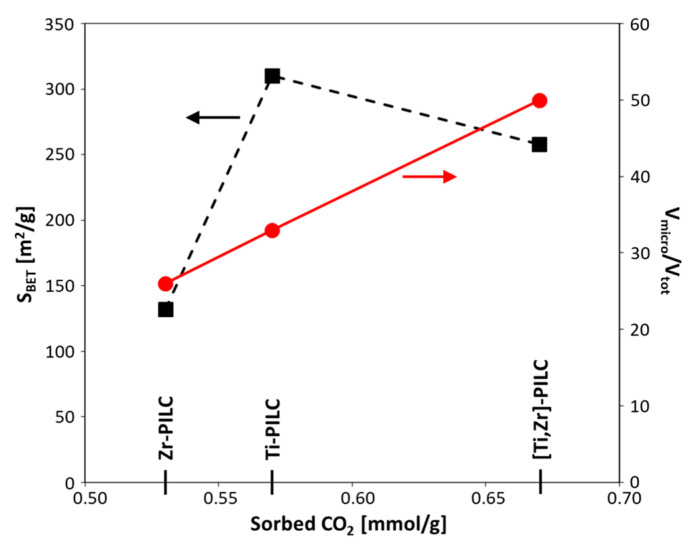
The amount of sorbed CO_2_ versus specific surface area and relative share of micropores in pillared montmorillonites.

The values of CO_2_ sorption at 0 °C and atmospheric pressure (p/p_0_ = 0.03) are given in Table 3. It is clear that pillaring has an extremely beneficial effect on the ability of montmorillonite to capture CO_2_. At least a fourfold increase in the amount of sorbed CO_2_ when passing from raw bentonite to the pillared samples suggests that sorption took place mainly in the interlayer spaces made accessible by the pillaring. Differences between the amounts of CO_2_ adsorbed on various pillared montmorillonites were relatively low. Noteworthy, the highest sorption was found for the Ti–Zr-pillared sample, which showed a specific surface area about 50 m^2^/g lower than that of Ti-PILC but displayed the highest relative share of micropores. Such a result implied that it was the microporosity, rather than the specific surface area, that controlled the sorption properties of pillared samples (Figure 7). Similar relationships were observed for low-pressure CO_2_ sorption on activated carbons [10,11,12,14,16,17,38,40].

It should be noted that while microporous characteristics of pillared clays are undoubtedly beneficial for CO_2_ capture, their acidic properties, associated with the presence of protons acting as species compensating the layer charge, are, in principle, a disadvantageous factor. The CO_2_ molecule is acidic itself; therefore, acid-functionalized surfaces are unfavorable to CO_2_ adsorption [42]. However, the protons can be exchanged for other cationic species, which opens the path to further modification of PILC, e.g., by exchange with inorganic or organic cationic species with basic character [43].

It is of interest to compare the performance of pillared sorbents prepared in this study from industrial-grade Kopernica bentonite with the previously published results obtained under similar conditions (0 °C, 1 bar) for various pillared montmorillonites. The data gathered in Table 4 show that a direct comparison can be conducted only in the case of Zr-PILC solids. It can be seen that the literature data on the CO_2_ capture ability of Zr-PILC sorbents obtained from different parent clays range from 0.5 to 1.5 mmol/g. The CO_2_ amount adsorbed by the Zr-PILC tested in the current study is at the level of the lowest value reported in the literature. However, it should be borne in mind that in previous works, the parent clays were subjected to preliminary purification, Na^+^ exchange, or both. In contrast, the starting material used in this study was crude industrial bulk bentonite. This shows that such a cost-effective approach to the preparation of PILC sorbents is a viable option.

**Figure 8:**
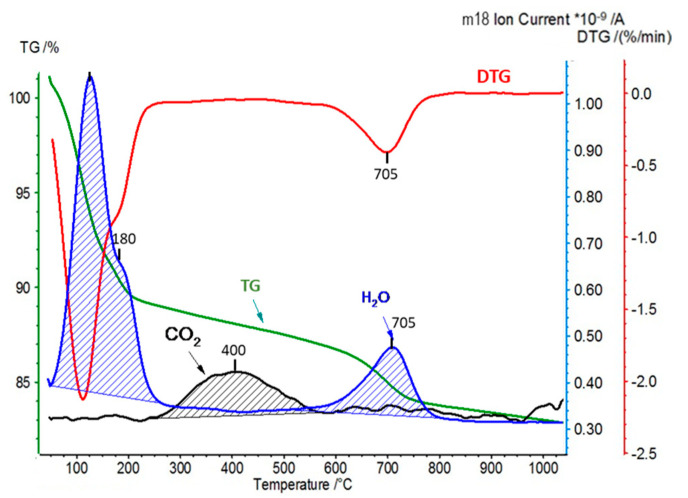
STA/QMS analysis of Kopernica bentonite ((for better visibility, the CO_2_ (*m/z* = 44) trace has been magnified and is not on scale).

In the second experimental approach, the interaction of pillared montmorillonite derivatives with carbon dioxide was carried out in a flow system operating at room temperature. The amounts of CO_2_ retained by the materials under such conditions were determined by subjecting the samples to the simultaneous thermal analysis (STA) and the quadrupole mass spectrometry (QMS) analysis of the evolving gases (CO_2_ and H_2_O). STA/QMS analysis of the Kopernica parent material was also performed for reference purposes (Figure 8).

The TG/DTG curves, together with the profile of H_2_O (*m/z* = 18) evolution, were consistent with the thermal decomposition of Ca-montmorillonite, as discussed in detail in [35]. The TG curve showed two major weight losses. The first one, below 250 °C, was associated with the departure of water hydrating Ca^2+^ cations. The water of hydration, forming a double coordination sphere around Ca^2+^, was released in two steps, visible as overlapping effects in DTG trace and H_2_O QMS signal. The second weight loss, associated with DTG minimum at 700 °C and the corresponding maximum on the H_2_O QMS profile, was due to the dehydroxylation of montmorillonite layers. QMS analysis revealed that, besides water, CO_2_ (*m/z* = 44), absorbed from the atmosphere, was also released from the sample, albeit its signal was nearly two orders of magnitude less intense. The maximum CO_2_ evolution appeared at a relatively high temperature of 400 °C, which showed that chemisorption rather than physisorption was responsible for the carbon dioxide bound in montmorillonite. The most likely sites of CO_2_ chemisorption are the basic sites at the layer edges, such as basic hydroxyl groups or basic surface oxygens, yielding various carbonate-like forms upon interaction with CO_2_ [25,44,45]. The expulsion of CO_2_ did not overlap with the release of water of hydration, indicating that no meaningful dissolution/reaction of CO_2_ with water occurred.

**Figure 9:**
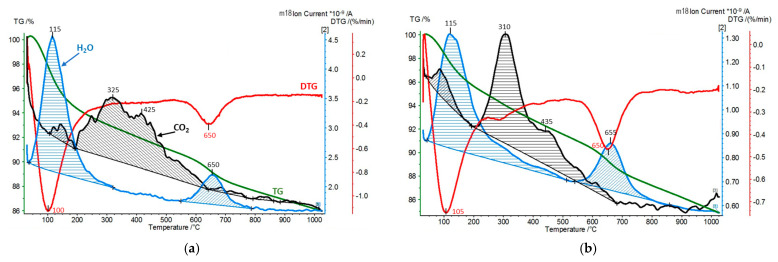
STA/QMS analysis for Zr-PILC: (**a**) as received and (**b**) after 24 h exposure to CO_2_ flow at room temperature (for better visibility, the CO_2_ (*m/z* = 44) traces have been magnified and are not in scale).

**Figure 10:**
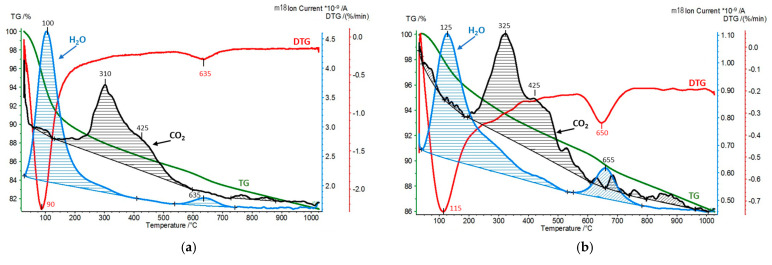
STA/QMS analysis for Ti-PILC: (**a**) as received and (**b**) after 24 h exposure to CO_2_ flow at room temperature (for better visibility, the CO_2_ (*m/z* = 44) traces have been magnified and are not in scale).

**Figure 11:**
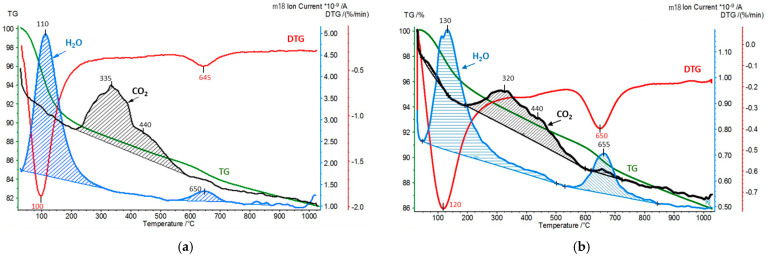
STA/QMS analysis for [Ti,Zr]-PILC: (**a**) as received and (**b**) after 24 h exposure to CO_2_ flow at room temperature (for better visibility, the CO_2_ (*m/z* = 44) traces have been magnified and are not in scale).

Figure 9, Figure 10 and Figure 11 show the results of STA/QMS analysis performed for Zr-PILC, Ti-PILC, and [Ti,Zr]-PILC samples, respectively, before and after 24-h exposure to dry CO_2_ flow at room temperature. For better visibility, the CO_2_ traces (*m*/*z* = 44) have been magnified and are not in scale. Quantitative information on the amount of adsorbed CO_2_ derived from integrated peak areas is given in Table 5, which also shows the amount of released H_2_O corresponding to hydration water loss and dehydroxylation, as determined from TG curves.

It is worth noting that the amount of chemisorbed CO_2_ removed from the raw Kopernica bentonite was about twice as high as the amount removed from the pillared samples. As mentioned earlier, pillared montmorillonites are acidic materials due to the presence of protons in their structure, compensating for the layer charge. For this reason, their surface becomes less susceptible to CO_2_ adsorption. In addition, due to the acidic reaction environment during pillaring, there was a leaching of octahedral cations from the edges of the montmorillonite layers, thus reducing the number of potential CO_2_ chemisorption sites.

Analysis of Figure 9, Figure 10 and Figure 11 revealed a series of effects common to all the materials tested, resulting from pillaring and prolonged exposure to CO_2_ flow.

First, a comparison with Figure 8 showed that in all the as-received pillared clays, a build-up of evolving CO_2_ maximum at 310–325 °C was observed. The maximum overlapped with a second broad maximum, centered around 420–440 °C. The latter, occurring in a temperature range close to that observed for CO_2_ desorption from untreated Kopernica, can be assigned to the desorption of CO_2_ from the edges of montmorillonite layers. As for the other maximum, it is reasonable to attribute it to CO_2_ chemisorbed in the interlayer spaces opened up by pillaring, most likely at the oxide pillars.

Second, in all PILC samples, the first water loss tailed off toward higher temperatures and partially overlapped with the evolution of CO_2_. The effect became even more pronounced after the treatment of PILC samples in CO_2_ flow, where the end of low-temperature water loss coincided with the onset of the structural dehydroxylation effect. The simultaneous evolution of water and CO_2_ may be taken as an indication that at least some of the CO_2_ was chemisorbed in the form of bicarbonates.

Third, the effect of dehydroxylation (around 650 °C), which was rather weak in pillared clays due to thermal treatment during preparation, gained in intensity after CO_2_ flow treatment (Table 2). Moreover, in the case of Ti-PILC and [Ti,Zr]-PILC, and to a much lesser extent in the case of Zr-PILC, the total amount of adsorbed CO_2_ diminished after treatment in CO_2_ flow, as did the amount of water of hydration responsible for the low-temperature emission of H_2_O (Table 2). Seeking to substantiate these observations, it should be recalled that treatment with dry CO_2_ creates conditions that allow water to be lost from the sample. The amount of water in hydration depends strongly on the relative humidity. The flow of dry CO_2_ creates a water-free atmosphere over the sample, and adjustment to the new conditions involves the loss of some water of hydration. The fact that Zr-PILC is relatively immune to the change in the atmosphere is most likely due to the very low interlayer distance in this sample, which hinders the diffusion of water from the structure.

Furthermore, the primary role of water hydration in PILC samples is to interact with protons through the formation and hydration of hydronium ions. The partial loss of water of hydration means that the protons stripped of their hydration shell can migrate through the structure and, as strongly acidic species, can effectively compete with CO_2_ for basic chemisorption sites, causing some of the chemisorbed CO_2_ to be expelled [46]. Migrating protons can also be trapped by basic oxygen ions, leading to the regeneration of hydroxyl groups and, consequently, to increased water loss associated with the dehydroxylation of montmorillonite.

## 4. Conclusions

Investigation of CO_2_ sorption at 0 °C and 1 bar pressure over Ti-, Zr, and [Ti,Zr]-pillared montmorillonites showed at least a 4-fold increase of gas sorption on PILC samples compared to the raw parent material. The highest CO_2_ sorption was found for mixed [Ti,Zr]-pillared montmorillonite, which also showed the highest share of micropores in total pore volume. This factor clearly favored CO_2_ sorption under the conditions of the experiment conducted.

Exposure of PILC samples to a flow of dry CO_2_ modified both the sorption and the structural characteristics of the PILC sorbents, especially in the case of Ti- and [Ti,Zr]-pillared montmorillonites, with larger interlayer distances. Among other things, it was observed that (a) the amount of CO_2_ retained by the sorbents decreased, and (b) the amount of structural hydroxyls increased. The effects were attributed to the ability of protons, present in PILC samples as species compensating the layer charge, to migrate through the structure and effectively compete with CO_2_ for the basic chemisorption sites and/or to react with basic lattice oxygens to form hydroxyls.

Based on CO_2_ sorption experiments and the study of the interaction of PILCs with dry CO_2_, further modification of supported montmorillonites by exchanging protons for inorganic or organic base cations can be proposed as a worthwhile way to further improve the CO_2_ capture capacity of these systems.

## Figures and Tables

**Table 1:** Chemical composition of investigated samples.

Sample	SiO_2_ [wt.%]	Al_2_O_3_ [wt.%]	MgO [wt.%]	Fe_2_O_3_ [wt.%]	CaO [wt.%]	Na_2_O [wt.%]	K_2_O [wt.%]	TiO_2_ [wt.%]	ZrO_2_ [wt.%]
Kopernica	70.95	21.04	2.71	2.37	2.09	0.06	0.58	0.16	0.05
Zr-PILC	59.95	19.30	2.02	2.01	0.04	0.05	0.40	0.13	16.08
Ti-PILC	43.63	13.31	1.36	1.42	0.07	0.04	0.28	39.76	0.03
[Ti,Zr]-PILC	53.54	17.33	1.86	1.78	0.04	0.06	0.29	9.41	15.64

**Table 2:** XRD 001 reflections of the parent Kopernica montmorillonite and samples obtained by cation exchange with Zr, Ti, and [Ti,Zr] pillaring species before and after calcination at 400 °C.

Sample	001 Reflection Maximum [° 2θ]	001 Reflection FWHM [° 2θ]	d_001_ [nm]
Kopernica	6.03	1.20	1.47
Zr-Mt	4.70	0.94	1.88
Zr-PILC	5.38	1.17	1.64
Ti-Mt	3.55	0.92	2.49
Ti-PILC	3.94	0.87	2.24
[Ti,Zr]-Mt	4.25	1.00	2.08
[Ti,Zr]-PILC	4.55	1.07	1.94

**Table 3:** Textural parameters from N_2_ adsorption/desorption isotherms at −196 °C (S_BET_—BET specific surface area, V_tot_—total pore volume, V_mic_—micropore volume, V_meso_—mesopore volume, V_macro_—macropore volume, D_av_—average pore size, d_001_—basal interplanar distance from XRD measurement, CO_2_—amount of sorbed CO_2_ at 0 °C and p/p_0_ = 0.03).

Sample	S_BET_ [m^2^/g]	V_tot_ [cm^3^/g]	V_micro_ [cm^3^/g]	V_micro_/V_tot_	V_meso_ [cm^3^/g]	V_meso_/V_tot_	V_macro_ [cm^3^/g]	V_macro_/V_tot_	D_av_ [nm]	d_001_ [nm]	CO_2_ [mmol/g]
Kopernica	52	0.17	0.02	0.12	0.11	0.65	0.04	0.23	13.2	1.47	0.16
Zr-PILC	132	0.21	0.06	0.26	0.09	0.45	0.06	0.29	6.3	1.65	0.53
Ti-PILC	310	0.36	0.12	0.33	0.17	0.46	0.07	0.20	4.7	2.24	0.57
[Ti,Zr]-PILC	258	0.23	0.12	0.50	0.09	0.37	0.03	0.12	3.6	1.94	0.67

**Table 4:** The literature data on CO_2_ adsorption capacity at 1 bar and 0 °C of pillared montmorillonite/bentonite materials (rounded off to the decimal places), with an indication of the clay provenience and pretreatment.

Pillared Clay	Clay Provenience/ Pretreatment	CO_2_ Adsorption Capacity (mmol CO_2_/g)	Reference
Al-PILC	Montmorillonite Tsukinuno, Japan/ purified	1.0	[23]
Al-PILC	Montmorillonite Kunipia, Japan/ Na-exchanged	0.8	[24]
Al-PILC	Montmorillonite Wyoming, USA/ Na-exchanged	0.6	[24]
Zr-PILC	Montmorillonite Tsukinuno, Japan/ purified	0.8	[23]
Zr-PILC	Bentonite Benavila-Alentejo, Portugal/ purified, Na-exchanged	1.5	[20]
Zr-PILC	Montmorillonite Wyoming, USA/ Na-exchanged	0.5	[24]
(TiO_2_–SiO_2_)-PILC	Montmorillonite Wyoming, USA/ Na-exchanged	1.2	[24]
Zr-PILC	Industrial bentonite Kopernica, Slovakia/ as received	0.5	This work
Ti-PILC	Industrial bentonite Kopernica, Slovakia/ as received	0.6	This work
[Ti,Zr]-PILC	Industrial bentonite Kopernica, Slovakia/ as received	0.7	This work

**Table 5:** Selected STA/QMS parameters of parent Kopernica bentonite and Zr-, Ti-, and [Zr,Ti]-pillared montmorillonites: amount of desorbed CO_2_ (from QMS), weight loss below 250 °C attributed to the departure of water of hydration (TG), and weight loss due to dehydroxylation.

Sample	CO_2_ Desorption/QMS [mmol/g]	H_2_O Hydration/TG [wt.%]	H_2_O Dehydroxylation/TG [wt.%]
Kopernica	0.10	12.0	4.0
Zr-PILC	0.04	6.3	2.9
Zr-PILC exposed to dry CO_2_	0.03	5.0	4.0
Ti-PILC	0.04	11.1	2.4
Ti-PILC exposed to dry CO_2_	0.02	5.1	3.0
[Ti,Zr]-PILC	0.05	10.8	3.1
[Ti,Zr]-PILC exposed to dry CO_2_	0.01	5.0	4.0

## Data Availability

The data presented in this study are available on request from the authors.

## References

[1] Bergaya F., Lagaly G. (2013). General Introduction: Clays, Clay Minerals, and Clay Science. Developments in Clay Science.

[2] Borah D., Nath H., Saikia H. (2022). Modification of bentonite clay & its applications: A review. Rev. Inorg. Chem..

[3] Starý J., Jirásek J., Pticen F., Zahradník J., Sivek M. (2021). Review of production, reserves, and processing of clays (including bentonite) in the Czech Republic. Appl. Clay Sci..

[4] Abdulkareem M.A., Al-Kaabi F.S., Muhsin N.A., Badr N.D., Radhi D.J. (2024). Activation of calcium bentonite for use as a drilling fluid: Physical and chemical methods evaluation. Case Stud. Chem. Environ. Eng..

[5] Vicente M.A., Gil A., Bergaya F., Bergaya F., Lagaly G. (2013). Pillared Clays and Clay Minerals. Developments in Clay Science.

[6] Gil A., Vicente M.A. (2020). Progress and perspectives on pillared clays applied in energetic and environmental remediation processes. Curr. Opin. Green Sustain. Chem..

[7] Baloyi J., Ntho T., Moma J. (2018). Synthesis and application of pillared clay heterogeneous catalysts for wastewater treatment: A review. RSC Adv..

[8] Soo X.Y.D., Lee J.J.C., Wu W.Y., Tao L., Wang C., Zhu Q., Bu J. (2024). Advancements in CO_2_ capture by absorption and adsorption: A comprehensive review. J. CO_2_ Util..

[9] Kumar S., Srivastava R., Koh J. (2020). Utilization of zeolites as CO_2_ capturing agents: Advances and future perspectives. J. CO_2_ Util..

[10] Shi X., Xiao H., Azarabadi H., Song J., Wu X., Chen X., Lackner K.S. (2020). Sorbents for Direct Capture of CO_2_ from Ambient Air. Angew. Chem. Int. Ed..

[11] Kamran U., Park S.-J. (2021). Chemically modified carbonaceous adsorbents for enhanced CO_2_ capture: A review. J. Clean. Prod..

[12] Halliday C., Hatton T.A. (2021). Sorbents for the Capture of CO_2_ and Other Acid Gases: A Review. Ind. Eng. Chem. Res..

[13] Chang R., Wu X., Cheung O., Liu W. (2022). Synthetic solid oxide sorbents for CO_2_ capture: State-of-the art and future perspectives. J. Mater. Chem. A.

[14] Dziejarski B., Serafin J., Andersson K., Krzyżyńska R. (2023). CO_2_ capture materials: A review of current trends and future challenges. Mater. Today Sustain..

[15] Volzone C. (2007). Retention of pollutant gases: Comparison between clay minerals and their modified products. Appl. Clay Sci..

[16] Rehman A., Nazir G., Rhee K.Y., Park S.J. (2021). A rational design of cellulose-based heteroatom-doped porous carbons: Promising contenders for CO_2_ adsorption and separation. Chem. Eng. J..

[17] He S., Chen G., Xiao H., Shi G., Ruan C., Ma Y., Dai H., Yuan B., Chen X., Yang X. (2021). Facile preparation of N-doped activated carbon produced from rice husk for CO_2_ capture. J. Colloid Interface Sci..

[18] Baksh M.S.A., Yang R.T. (1992). Unique adsorption properties and potential energy profiles of microporous pillared clays. AIChE J..

[19] Molinard A., Vansant E.F. (1995). Controlled gas adsorption properties of various pillared clays. Adsorption.

[20] Pereira P.R., Pires J., Brotas de Carvalho M. (1998). Zirconium pillared clays for carbon dioxide/methane separation. 1. Preparation of adsorbent materials and pure gas adsorption. Langmuir.

[21] Gil A., Gandía L.M. (2003). Microstructure and quantitative estimation of the micropore-size distribution of an alumina-pillared clay from nitrogen adsorption at 77K and carbon dioxide adsorption at 273K. Chem. Eng. Sci..

[22] Peng X., Zhao J., Cao D. (2007). Adsorption of carbon dioxide of 1-site and 3-site models in pillared clays: A Gibbs ensemble Monte Carlo simulation. J. Coll. Interface Sci..

[23] Garcés-Polo S.I., Villarroel-Rocha J., Sapag K., Korili S.A., Gil A. (2016). A comparative study of CO_2_ diffusion from adsorption kinetic measurements on microporous materials at low pressures and temperatures. Chem. Eng. J..

[24] Wang K., Yan X., Komarneni S. (2018). CO_2_ Adsorption by Several Types of Pillared Montmorillonite Clays. Appl. Petrochem. Res..

[25] Chouikhi N., Cecilia J.A., Vilarrasa-García E., Besghhaier S., Chlendi M., Duro F.I.F., Castellon E.R., Bagane M. (2019). CO_2_ adsorption of materials synthesized from clay minerals: A review. Minerals.

[26] Wu K., Ye Q., Wu R., Dai H. (2020). Alkali metal-promoted aluminum-pillared montmorillonites: High-performance CO_2_ adsorbents. J. Solid State Chem..

[27] Wu K., Ye Q., Wu R., Chen S., Dai H. (2020). Carbon dioxide adsorption behaviors of aluminum-pillared montmorillonite-supported alkaline earth metals. J. Environ. Sci..

[28] Tao H., Qian X., Zhou Y., Cheng H. (2022). Research progress of clay minerals in carbon dioxide capture. Renew. Sustain. Energy Rev..

[29] Wal K., Rutkowski P., Stawinski W. (2021). Application of clay minerals and their derivatives in adsorption from gaseous phase. Appl. Clay Sci..

[30] Tingelinhas J., Saragoça C., Al Mohtar A., Mateus M., Pinto M.L. (2023). Pillared clays as cost-efective adsorbents for carbon capture by pressure swing adsorption processes in the cement industry. Ind. Eng. Chem. Res..

[31] Aneja R., Chauhan A., Chauhan T., Vyas R., Saini V.K. (2024). Understanding adsorption selectivity in zirconium-pillared clays for biogas upgradation: The role of metal/clay ratio. Environ. Sci. Pollut. Res..

[32] Bahranowski K., Włodarczyk W., Wisła-Walsh E., Gaweł A., Matusik J., Klimek A., Gil B., Michalik-Zym A., Dula R., Socha R.P. (2015). [Ti,Zr]-pillared montmorillonite-A new quality with respect to Ti- and Zr-pillared clays. Micropor. Mesopor. Mater..

[33] Michalik-Zym A., Dula R., Duraczyńska D., Kryściak-Czerwenka J., Machej T., Socha R.P., Włodarczyk W., Gaweł A., Matusik J., Bahranowski K. (2015). Active, selective and robust Pd and/or Cr catalysts supported on Ti-, Zr- or [Ti,Zr]-pillared montmorillonites for destruction of chlorinated volatile organic compounds. Appl. Catal. B Environ..

[34] Bahranowski K., Klimek A., Gaweł A., Górniak K., Michalik-Zym A., Serwicka-Bahranowska E. (2018). Structural transformations of hydrolysates obtained from Ti-, Zr-, and Ti, Zr-solutions used for clay pillaring: Towards understanding of the mixed pillars nature. Materials.

[35] Górniak K., Szydłak T., Gaweł A., Klimek A., Tomczyk A., Sulikowski B., Olejniczak Z., Motyka J., Serwicka E.M., Bahranowski K. (2016). Commercial bentonite from the Kopernica deposit (Tertiary, Slovakia): A petrographic and mineralogical approach. Clay Miner..

[36] González B., Pérez A.H., Trujillano R., Gil A., Vicente M.A. (2017). Microwave-Assisted Pillaring of a Montmorillonite with Al-Polycations in Concentrated Media. Materials.

[37] Pacula A., Bielanska E., Gaweł A., Bahranowski K., Serwicka E. (2006). Textural effects in powdered montmorillonite induced by freeze-drying and ultrasound pretreatment. Appl. Clay Sci..

[38] Thommes M., Kaneko K., Neimark A.V., Olivier J.P., Rodriguez-Reinoso F., Rouquerol J., Sing K.S.W. (2015). Physisorption of gases, with special reference to the evaluation of surface area and pore size distribution (IUPAC Technical Report). Pure Appl. Chem..

[39] Singh G., Lee J., Karakoti A., Bahadur R., Yi J., Zhao D., AlBahily K., Vinu A. (2020). Emerging trends in porous materials for CO_2_ capture and conversion. Chem. Soc. Rev..

[40] Zhang Z., Zhou J., Xing W., Xue Q., Yan Z., Zhuo S., Qiao S.Z. (2013). Critical role of small micropores in high CO_2_ uptake. Phys. Chem. Chem. Phys..

[41] Schoonheydt R.A., Johnston C.T., Bergaya F., Schoonheydt R., Johnston C.T., Bergaya F. (2018). Clay minerals and their surfaces. Surface and Interface Chemistry of Clay Minerals.

[42] Cecilia J.A., Vilarrasa-García E., Cavalcante C.L., Azevedo D.C.S., Franco F., Rodríguez-Castellón E. (2018). Evaluation of two fibrous clay minerals (sepiolite and palygorskite) for CO_2_ capture. J. Environ. Chem. Eng..

[43] Elkhalifah A.E.I., Maitra S., Bustam M.A., Murugesan T. (2013). Effects of exchanged ammonium cations on structure characteristics and CO_2_ adsorption capacities of bentonite clay. Appl. Clay Sci..

[44] Olszówka J.E., Karcz R., Bielańska E., Kryściak-Czerwenka J., Napruszewska B.D., Sulikowski B., Socha R.P., Gaweł A., Bahranowski K., Olejniczak Z. (2018). New insight into the preferred valency of interlayer anions in hydrotalcite-like compounds: The effect of Mg/Al ratio. Appl. Clay Sci..

[45] Li M., Tumuluri U., Wu Z., Dai S. (2015). Effect of Dopants on the Adsorption of Carbon Dioxide on Ceria Surfaces. ChemSusChem.

[46] Kashif M., Yuan M., Su Y., Heynderickx P.M., Memon A. (2023). A review on pillared clay-based catalysts for low-temperature selective catalytic reduction of NOx with hydrocarbons. Appl. Clay Sci..

